# Inferring causal phenotype networks using structural equation models

**DOI:** 10.1186/1297-9686-43-6

**Published:** 2011-02-10

**Authors:** Guilherme JM Rosa, Bruno D Valente, Gustavo de los Campos, Xiao-Lin Wu, Daniel Gianola, Martinho A Silva

**Affiliations:** 1 Department of Animal Sciences, University of Wisconsin - Madison, Madison, WI 53706, USA; 2 Department of Biostatistics & Medical Informatics, University of Wisconsin - Madison, Madison, WI 53706, USA; 3 Federal University of Minas Gerais, Belo Horizonte, MG 30123, Brazil; 4 Department of Biostatistics, University of Alabama at Birmingham, Birmingham, AL 35201, USA; 5 Department of Dairy Science, University of Wisconsin - Madison, Madison, WI 53706, USA

## Abstract

Phenotypic traits may exert causal effects between them. For example, on the one hand, high yield in dairy cows may increase the liability to certain diseases and, on the other hand, the incidence of a disease may affect yield negatively. Likewise, the transcriptome may be a function of the reproductive status in mammals and the latter may depend on other physiological variables. Knowledge of phenotype networks describing such interrelationships can be used to predict the behavior of complex systems, e.g. biological pathways underlying complex traits such as diseases, growth and reproduction. Structural Equation Models (SEM) can be used to study recursive and simultaneous relationships among phenotypes in multivariate systems such as genetical genomics, system biology, and multiple trait models in quantitative genetics. Hence, SEM can produce an interpretation of relationships among traits which differs from that obtained with traditional multiple trait models, in which all relationships are represented by symmetric linear associations among random variables, such as covariances and correlations. In this review, we discuss the application of SEM and related techniques for the study of multiple phenotypes. Two basic scenarios are considered, one pertaining to genetical genomics studies, in which QTL or molecular marker information is used to facilitate causal inference, and another related to quantitative genetic analysis in livestock, in which only phenotypic and pedigree information is available. Advantages and limitations of SEM compared to traditional approaches commonly used for the analysis of multiple traits, as well as some indication of future research in this area are presented in a concluding section.

## Background

In animal breeding and quantitative genetics, relationships among phenotypic traits are traditionally studied via probabilistic relationships between them, using standard Multiple Trait Models (MTM) - see, for example, [1,2]. Although such models can be used satisfactorily to infer how probable events are, they are not stable enough to predict how probabilities would change as a result of external interventions [3,4]. In biological systems, phenotypic traits may exert causal effects between them. For example, on the one hand, high yield in dairy cows may increase the liability to certain diseases and, on the other hand, the incidence of a disease may affect yield negatively. Likewise, the transcriptome may be a function of the reproductive status in mammals and the latter may depend on other physiological variables. Such phenotypic relationships can be studied using statistical models that account for recursiveness and feedback between traits.

Information regarding phenotype networks describing such interrelationships can be used to predict the behavior of complex systems, e.g. biological pathways underlying complex traits such as diseases, growth and reproduction, and ultimately it can be used to optimize management practices and multi-trait selection strategies in livestock. For instance, a correlation between traits *y*_1 _and *y*_2 _can be due to a direct effect of *y*_1 _on *y*_2 _(or *y*_2 _on *y*_1_) or to extraneous variables that jointly affect *y*_1 _and *y*_2_. Knowledge about the causal structure underlying phenotypic relationships is necessary to predict the effect of interventions (e.g., management practices) applied to trait *y*_1 _or *y*_2_. For example, if trait *y*_1 _affects *y*_2_, and *y*_2 _has no effect on *y*_1_, an intervention on *y*_1 _will cause changes on *y*_2_, but the reverse would not hold true.

**Figure 1:**
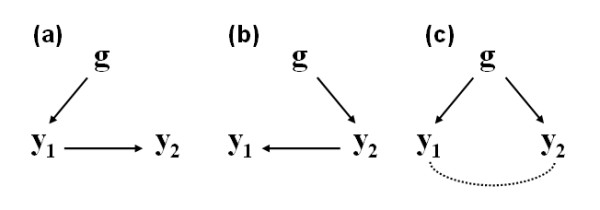
**Some possible gene-phenotype networks involving a single gene (*g*) and two phenotypic traits (*y*_1 _and *y*_2_)**. Standard multi-trait statistical models could potentially detect a correlation between the two phenotypic traits and a pleiotropic effect of gene *g*; however, only gene-phenotype network and causal models would be able to distinguish the paths connecting them

Similar situations can be considered from a genetic improvement standpoint. Conventionally, genetic correlation is defined as the proportion of variance that two traits share due to genetic causes, and it indicates how much of the genetic influence on two traits is common to both, e.g., due to pleiotropism. However, different scenarios can cause a pleiotropic effect of a specific gene (*g*) on two traits (*y*_1 _and *y*_2_), as illustrated in Figure 1: (a) the expression of the gene changes trait *y*_1_, and the phenotypic change on trait *y*_1 _affects trait *y*_2_; (b) the expression of the gene acts on trait y_2_, and the phenotypic changes on trait *y*_2 _modify trait *y*_1_; or (c) the expression of the gene changes both traits directly, which may or may not have a phenotypic causal effect between them. Knowledge about these different sources of genetic correlation between traits could be used to further improve selection decisions and increase the genetic progress of breeding programs.

As an alternative to the traditional MTM used in animal breeding and genetics, Structural Equation Models (SEM; [5,6]) can be applied to study recursive and simultaneous relationships among phenotypes in multivariate systems. Therefore, SEM can produce an interpretation of relationships among traits which differs from that obtained with standard MTM, where all relationships are represented by symmetric linear associations among random variables, i.e., as measured by covariances and correlations. Unlike MTM, in SEM one trait can be treated as a predictor of another trait, providing a functional (causal) link between them.

In the last few years, genetics has been used as a means to infer phenotype networks, including causal relationships among them [7], and SEM or related methodologies have been employed for such tasks (e.g., [8-12]). These applications of SEM to reconstruct phenotype networks considered genetical genomics studies with model species, using quantitative trait loci (QTL), molecular marker, and or DNA sequence information to facilitate causal inference. However, even with livestock, in which genetical genomics studies are not common due to its cost, and reliable information regarding QTL or even sequence information may not be available, SEM have also been satisfactorily used to study phenotypic networks. SEM within a quantitative genetics mixed models context have been described by [13]. Many authors have used such an approach (e.g., [14,15]), but typically the causal structures are pre-selected using some sort of prior knowledge. More recently, Valente et al. [16] have proposed a methodology that allows searching for recursive causal structures in the context of mixed models for the genetic analysis of multiple traits, showing that under certain conditions it may be possible to infer phenotype networks and causal effects even without QTL or marker information. In this paper, we briefly review SEM and present some of their applications for phenotype network reconstruction in genetical genomics studies, in which both phenotypic and molecular information is available, as well as in the context of classical quantitative genetic analysis of multiple phenotypic traits, using pedigree information.

### 1. Structural equation models

**Figure 2:**
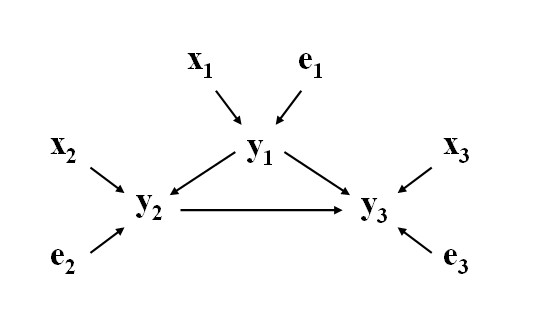
**Example of a causal structure, in which *y*'s represent measurements on three phenotypic traits, *x*'s and *e*'s represent known explanatory variables and residual factors affecting *y*'s, respectively**.

Structural Equation Models [3,4] provide a general statistical modeling technique to estimate and test functional relationships among traits, which are often not revealed by standard linear models. When fitting a SEM to a set of variables, it is necessary to define a priori, for each variable, the subset of the remaining variables that have a (direct) causal effect on it. This information is called 'causal structure', and can be represented as a directed graph in which variables (measured or unmeasured) constitute nodes and causal relationships are represented as directed edges between nodes. For example, consider the graph depicted in Figure 2, in which explanatory variables *x *and some additional (residual) variables *e *directly affect variables *y*, which have also some causal relationships among them.

The graph in Figure 2 can be represented by a set of structural equations, given by:

$$\left\{ \begin{array}{l} y_{1}=\beta_{1}x_{1}+e_{1}\\ y_{2}=\lambda_{21}y_{1}+\beta_{2}x_{2}+e_{2}\\ y_{3}=\lambda_{31}y_{1}+\lambda_{32}y_{2}+\beta_{3}x_{3}+e_{3} \end{array}\right.$$

where *β*'s are model parameters representing the "fixed effects" of the *x *covariates on *y*'s, and *λ*'s are structural coefficients representing the magnitude of the casual effects among *y*'s. Hence, in matrix notation, a SEM can be represented as **y **= **Λy **+ **Xβ **+ **e**, where **Λ **is a quadratic matrix with zeroes in the diagonal and with structural coefficients *λ *or zeroes in the off-diagonal, and **y**, **X**, **β **and **e **are appropriate vectors or matrices with the observations *y*'s, exogenous variables *x*'s, model parameters *β*'s and residuals *e*'s, respectively. Competing networks representing different causal structures among *y*'s may be compared using some model selection criteria, such as likelihood ratio tests (LRT), Akaike information criterion (AIC; [17]), Bayesian information criterion (BIC; [18]), or Bayesian model selection approaches (see, for example, [19]).

Structural equation models have been intensively used in many fields, such as economics, psychometrics, social statistics, and biological sciences. In genetics, they have been used, for example, to study the relationships between phenotypic traits in humans, especially in the context of twin designs (e.g. [20,21]). More recently, it has been also employed in quantitative genetics mixed model analysis, and on gene-phenotype network reconstruction, as discussed below.

### 2. QTL information and the randomization of alleles

Thomas and Conti [22] have pointed out that genetically randomized experimental populations that segregate naturally occurring allelic variants can provide a basis for the inference of networks of causal associations among genetic loci, physiological phenotypes, and disease states. In particular, the randomization of alleles that occurs during meiosis provides a setting that is analogous to a randomized experimental design, such that causality can be inferred within the classical Fisherian statistical framework.

In this context, Schadt et al. [7] have proposed a multi-step procedure to infer causal relationships between two phenotypic traits and a common QTL. More specifically, they have tried to disentangle the causal path involving the expression of a particular gene, a cis-acting expression QTL (eQTL), and a complex trait (e.g. a disease trait), to determine if they are related to each other following a causal, reactive or independent model. Such models (denoted here as Models C, R and I, respectively) can be represented as in Figure 1, in which the variables *g*, *y*_1 _and *y*_2 _denote the cis-acting eQTL, the transcriptional activity of the gene, and the complex trait, respectively. Model C depicted in Figure 1a refers to the simplest causal relationship with respect to *y*_1_, in which allelic variations in *g *change *y*_2 _by changing the transcriptional activity *y*_1_. Model R (Figure 1b) represents the simplest reactive model with respect to *y*_1_, in which the expression *y*_1 _is modulated by the trait *y*_2_. Lastly, Model I (Figure 1c) represents a situation in which the QTL *g *controls *y*_1 _and *y*_2 _independently.

Schadt et al. [7] have proposed a likelihood-based causality model selection (LCMS) test that uses conditional correlation measures to determine which relationship among a trio of traits (a transcriptional trait, a complex phenotype, and a common QTL affecting both) is best supported by the data. Likelihoods associated with each of the models (causal, reactive and independent models) have been constructed and maximized with respect to the model parameters, and the AIC criterion has been used to select the model best supported by the data. More specifically, the joint probability distributions of the three models depicted in Figure 1 have been described as:

$$\{\begin{array}{c} {\text{M}}_{\text{C}}:p(g,y_{1},y_{2})=p(g)p(y_{1}|g)p(y_{2}|y_{1})\\ {\text{M}}_{\text{R}}:p(g,y_{1},y_{2})=p(g)p(y_{2}|g)p(y_{1}|y_{2})\\ {\text{M}}_{\text{I}}:p(g,y_{1},y_{2})=p(g)p(y_{1}|g)p(y_{2}|g,y_{1}) \end{array}$$

where *y*_1 _and *y*_2 _were assumed normally distributed about each genotypic mean at the common locus *g*. With those settings, model-specific likelihoods were obtained and standard maximum likelihood estimation methods have been employed.

Schadt et al. [7] have applied their methodology to a mouse genetical genomics study comprised of large-scale genotypic, gene-expression and complex-trait data to identify genes related to obesity, and have been able to identify known and new susceptibility genes for fat mass, and to successfully predict transcriptional response to perturbation in such genes. Their procedure, however, is restricted to simple gene-phenotypes networks, focusing on the identification of genes in the causal-reactive interval considering a trio of nodes comprising a common QTL affecting the expression of a specific gene and a complex trait. Evidently, gene and phenotype networks can be much more complex, as the causal-reactive genes may be also interacting in a broader network through an intricate cascade of genes and phenotypic traits.

**Figure 3:**
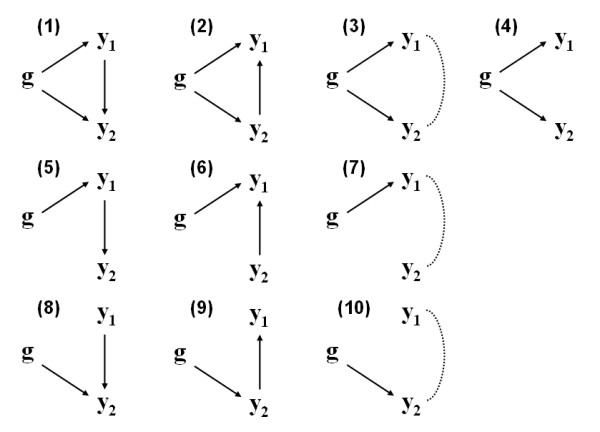
**Causal relationships among a QTL (*g*) and two correlated phenotypes (*y*_1 _and *y*_2_)**. Arrows indicate the direction of causal effects and dotted lines represent unresolved associations between the two phenotypes (adapted from [8])

More specifically with SEM, Li et al. [8] have presented a methodology to analyze multilocus, multitrait genetic data. Their method extends that of [7], not only by the number of loci and phenotypic traits studied, but also by different possible causal relationships among them, such that it provides a better characterization of the genetic architecture underlying complex traits. For instance, even if only a single locus and two correlated traits are considered, it allows for alternative recursive effects between phenotypes (Figure 3), outside the causal-reactive interval explored by [7].

The method of [8] comprises a series of five steps. First, single locus genome scans are run for each individual phenotype using a LOD-based test. Next, conditional genome scans are performed using one trait as a covariate in the analysis of another trait. As the authors mention, the choice of which trait(s) to use as covariates can be performed extensively or, alternatively, it may be guided by known biological relationships among the traits. In this setting, traits that are known to be upstream in the causal pathways should be employed as conditioning variables. The comparison between results from unconditioned and conditioned scans can give a first insight into the causal relationships among the phenotypes. For example, in model (8) of Figure 3, *g *and *y*_1 _are unconditionally independent; however, conditioning on *y*_2 _will result in a nonzero partial correlation between them. By contrast, in model (9), *g *and *y*_1 _are unconditionally correlated, and by conditioning on *y*_2 _their dependence vanishes. When the QTL *g *and both traits *y*_1 _and *y*_2 _are causally connected, as in model (1)-(3), the raw and partial correlations between them will all be nonzero, but they will change in magnitude depending on the signs of the path coefficients [8]. A third step on Li et al.'s [8] procedure refers to the construction of an initial path model and its respective SEM representation. In the graphical SEM, each measured trait is represented as a node, including the QTL identified in steps 1 and 2. Edges should be directed from the QTL to the corresponding traits, and edges should be added also from conditioning traits to the responses whenever a significant difference in LOD scores (ΔLOD) is observed. After the path models are constructed, they are assessed in terms of goodness-of-fit by comparing the predicted and observed covariance matrices and by significance tests for individual path coefficients. Finally, an additional step is performed to refine the model, by proposing and assessing alternative models, which are generated by adding or removing edges in the initial model, or by reversing the causal direction of an edge. The authors use a LRT approach to compare such models, but they also suggest that alternative model criteria could be used, such as the AIC or variations thereof, or predictive ability assessed through some cross-validation strategy. Steps 4 and 5 of model refinement and assessment may be also carried iteratively.

Li et al. [8] have carried out the genome scans with tests on every 2 cM using a permutation approach, followed by the SEM component of the analysis. They have applied the methodology proposed to the analysis of body weight and weights of the inguinal, gonadal, peritoneal, and mesenteric fat pads of a SM × NZB intercross population with 260 females and 253 male mice raised on an atherogenic diet, and concluded that SEM provide an insightful descriptive approach to the genetic analysis of multiple traits, allowing the characterization of pleiotropic and heterogeneous genetic effects of multiple loci on multiple traits, as well as the physiological interactions among traits.

Another application of SEM for phenotype causal network inference has been presented by [9], who propose a methodology to search for a set of sparser structures within a putative directed network of causal regulatory relationships among gene expression levels and eQTL in genetical genomics studies. Their method encompasses three steps. First, eQTL mapping techniques are used to identify chromosomal regions modulating the expression of genes. Secondly, regulator-target pairs are identified, such that a directed network can be obtained. Finally, sparser optimal networks are sought within the initial directed network using a SEM approach. Liu et al. [9] have applied their methodology to a genetical genomics data on yeast containing information on expression levels of 4589 genes and genotypes for 2956 markers on 112 haploid offspring originating from a cross between a laboratory and a wild strain. They have detected a number of *cis*- and *trans*-acting eQTL and regulator-target pairs, from which a directed network comprising 28K+ regulator-target pairs was constructed. Based on a partition of this initial network, which comprises 168 genes involved in a cycle genes and all genes connected to the cycle genes by up to three edges and all the eQTL associated with these genes, a SEM analysis has been performed for its sparsification. The preliminary sub-network had 265 genes, 241 QTL, 832 edges connecting genes, and 640 edges connecting eQTL to genes. The resulting SEM network contained 475 edges connecting genes, and 468 edges connecting eQTL to genes. Some additional analyses have been performed to check for lists of genes with specific biological functions that were enriched on this network, revealing for example that 41.6% of the genes are involved in catalytic activity, and other 18% are involved in hydrolase activity.

**Figure 4:**
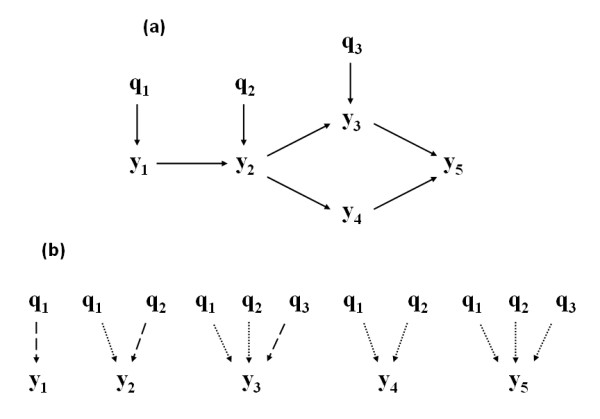
**Example of network with five phenotypes and three QTL (Panel a), and the expected output of single-trait QTL analyses for such phenotypes (Panel b)**. On Panel b, dashed and pointed arrows represent direct and indirect effects of QTLs on phenotypes, respectively

Also using QTL information to orient edges connecting phenotypes, Chaibub Neto et al. [11] have proposed a methodology comprised of two main steps. First, an association network is constructed using either an undirected dependency graph (UDG; [4]) or a skeleton derived from the PC algorithm of Spirtes et al. [23]. Second, LOD score tests are used to determine causal direction for every edge that connects a pair of phenotypes, conditional on QTL affecting the phenotypes. They have assessed the performance of their methodology in simulations studies, showing that it can recover network edges and infer their causal direction correctly at a high rate. However, although their method can be applied to human studies and outbred populations, it depends heavily on the availability of reliable information regarding QTL affecting the phenotypic traits of interest. Nonetheless, as discussed by [12], traditional QTL mapping approaches are based on single-trait analyses, in which the network structure among phenotypes is not taken into account. Such single-trait analyses may detect QTL that directly affect each phenotype, as well as QTL with indirect effects, which directly affect phenotypes upstream to the specific phenotype being analyzed. For example, consider the causal graph depicted in Figure 4a, consisting of five phenotypes (*y*_1_-*y*_5_) and three QTL (*q*_1_-*q*_5_). The outputs of single-trait analyses under this scenario are given in Figure 4b. Now, when a multi-trait QTL analysis is performed according to the actual phenotype causal network, detecting indirect-effect QTL is avoided by simply performing mapping analysis of each phenotype conditional on their parents (i.e., upstream phenotypes). For example, in Figure 4a, if a QTL analysis for phenotype *y*_3 _is performed conditionally on trait *y*_2_, only QTL *q*_3 _will be detected because *y*_3 _is conditionally orthogonal to *q*_1 _and *q*_2_, the two QTL with indirect effects (through *y*_1 _and *y*_2_) on *y*_3_.

Hence, traditional QTL mapping approaches that ignore the phenotype network result in poorly estimated genetic architecture of phenotypes, which may hamper correct inferences regarding causal relationships among phenotypes. In view of this drawback of traditional QTL analyses and phenotype network reconstruction methods, Chaibub et al. [12] have suggested a methodology that simultaneously infers a causal phenotype network and its associated genetic architecture. Their approach is based on jointly modeling phenotypes and QTL using homogeneous conditional Gaussian regression models and a graphical criterion for model equivalence. The concept of randomization of alleles during meiosis and the unidirectional relationship from genotype to phenotype are used to infer causal effects of QTL on phenotypes. Subsequently, causal relationships among phenotypes are inferred using the QTL nodes, which might make it possible to distinguish among phenotype networks that would otherwise be distribution equivalent.

### 3. Inferring causal phenotype networks with no genomic information

All phenotype network reconstruction approaches discussed so far rely on information regarding QTL affecting the phenotypes, or on the availability of genetic marker information for the joint inference regarding phenotype network and genetic architecture. Such QTL are used as parent nodes on putative networks, facilitating inferences on the remainder of the network, either on the construction of preliminary undirected graphs or on the establishment of causal relationships.

However, SEM have also been used to study relationships among phenotypic traits in the context of classical quantitative genetics and animal breeding, even if molecular marker or QTL information is not available. A methodology to insert SEM within a mixed effects model applied to quantitative genetics has been described by [13], and since then applied by many researchers working with different species and phenotypic traits. Some details regarding this methodology and examples of application are described below.

#### SEM embedded within a quantitative genetics mixed model

A SEM with a specific causal structure and random additive genetic effects can be written as [13,24]:

$${\text{y}}_{i}=\Lambda {\text{y}}_{i}+{\text{X}}_{i}\beta +{\text{u}}_{i}+{\text{e}}_{i},$$

where **y**_*i *_is a (*t *× 1) vector of phenotypic records on subject *i*; **Λ **is a (*t *× *t*) matrix of structural coefficients describing the chosen causal structure; **X**_*i*_**β **represents the effects of exogenous covariates as linear regressions, in which the matrix **X**_*i *_contains the covariates and **β **is a vector of 'fixed' regression coefficients; **u**_*i *_and **e**_*i *_are (*t *× 1) vectors of random additive genetic effects and model residuals, respectively, which are both associated with the *i*^*th *^subject. Furthermore, **u**_*i *_and **e**_*i *_are assumed to be distributed as $\left[\begin{array}{c} {\text{u}}_{i}\\ {\text{e}}_{i} \end{array}\right]\sim N\left\{\left[\begin{array}{c} 0\\ 0 \end{array}\right],\left[\begin{array}{cc} {\text{G}}_{0} & 0\\ 0 & \Psi_{0} \end{array}\right]\right\}$, where **G**_0 _and **Ψ**_0 _are the additive genetic and residual covariance matrices, respectively.

The model for *n *animals can be described as **y **= *(***Λ**⊗**I**_*n*_*)***y **+ **Xβ **+ **Zu **+ **e**, with:

$$\left[\begin{array}{c} \text{u}\\ \text{e} \end{array}\right]\sim N\left\{\left[\begin{array}{c} 0\\ 0 \end{array}\right],\left[\begin{array}{cc} {\text{G}}_{0}⊗\text{A} & 0\\ 0 & \Psi_{0}⊗{\text{I}}_{n} \end{array}\right]\right\},$$

where **y**, **u **and **e **are, respectively, vectors of phenotypic records, additive genetic effects and model residuals sorted by trait and subject within trait, and **X **and **Z **are incidence matrices relating effects in β and **u **and **y**. This model may be rewritten as [**I**_*tn*_-*(***Λ**⊗**I**_*n*_*)*] **y **= **Xβ **+ **Zu **+ **e**, so that an equivalent reduced model can be obtained as [13]:

$$\begin{array}{c} \text{y}={\left[{\text{I}}_{tn}-(\Lambda ⊗{\text{I}}_{n})\right]}^{-1}\text{X}\beta \\ +{\left[{\text{I}}_{tn}-(\Lambda ⊗{\text{I}}_{n})\right]}^{-1}\text{Z}\text{u}+{\left[{\text{I}}_{tn}-(\Lambda ⊗{\text{I}}_{n})\right]}^{-1}\text{e}. \end{array}$$

The resulting sampling distribution of **y **given the location parameters and the residual covariance matrix is:

$$\begin{array}{l} p(\text{y}|\Lambda ,\beta ,\text{u},\Psi_{0})\sim N\{{\left[{\text{I}}_{tn}-(\Lambda ⊗{\text{I}}_{n})\right]}^{-1}(\text{X}\beta +\text{Z}\text{u}),\\ \;{\left[{\text{I}}_{tn}-(\Lambda ⊗{\text{I}}_{n})\right]}^{-1}\Psi [{\text{I}}_{tn}-(\Lambda ⊗{\text{I}}_{n})]{'}^{-1}\} \end{array}$$

where **Ψ **= **Ψ**_0_⊗**I**_*n*_.

By reducing the SEM, the location and dispersion parameters are transformed into parameters of a standard MTM [24,25], as indicated below:

$$\begin{array}{c} {\text{y}}_{i}={\left({\text{I}}_{t}-\Lambda \right)}^{-1}{\text{X}}_{i}\beta \\ +{\left({\text{I}}_{t}-\Lambda \right)}^{-1}{\text{u}}_{i}+{\left({\text{I}}_{t}-\Lambda \right)}^{-1}{\text{e}}_{i}\\ =\mu_{i}^{*}+{\text{u}}_{i}^{*}+{\text{e}}_{i}^{*}, \end{array}$$

where $\mu_{i}^{*}={\left({\text{I}}_{t}-\Lambda \right)}^{-1}{\text{X}}_{i}\beta$, ${\text{u}}_{i}^{*}={\left({\text{I}}_{t}-\Lambda \right)}^{-1}{\text{u}}_{i}$, and ${\text{e}}_{i}^{*}={\left({\text{I}}_{t}-\Lambda \right)}^{-1}{\text{e}}_{i}$. In addition, the joint distribution of ${\text{u}}_{i}^{*}$ and ${\text{e}}_{i}^{*}$ is:

$$\left[\begin{array}{c} {\text{u}}_{i}^{*}\\ {\text{e}}_{i}^{*} \end{array}\right]\sim N\left(\left[\begin{array}{c} 0\\ 0 \end{array}\right],\left[\begin{array}{cc} {\text{G}}_{0}^{*} & 0\\ 0 & {\text{R}}_{0}^{*} \end{array}\right]\right),$$

with ${\text{G}}_{0}^{*}={\left({\text{I}}_{t}-\Lambda \right)}^{-1}{\text{G}}_{0}{\left({\text{I}}_{t}-\Lambda \right)}^{'-1}$ and ${\text{R}}_{0}^{*}={\left({\text{I}}_{t}-\Lambda \right)}^{-1}\Psi_{0}{\left({\text{I}}_{t}-\Lambda \right)}^{'-1}$.

Here, $\mu_{i}^{*}$, ${\text{u}}_{i}^{*}$, ${\text{e}}_{i}^{*}$, ${\text{G}}_{0}^{*}$ and ${\text{R}}_{0}^{*}$ are respectively the vectors of fixed effects, additive genetic effects, model residuals, and the genetic and residual covariance matrices of an MTM. Hence, it is seen that SEM and MTM are equivalent models, i.e.:

$$\begin{array}{l} N\left(\mu_{i}^{*}+{\text{u}}_{i}^{*},{\text{R}}_{0}^{*}\right)\\ \;=N\left({\left({\text{I}}_{t}-\Lambda \right)}^{-1}{\text{X}}_{i}\beta +{\left({\text{I}}_{t}-\Lambda \right)}^{-1}{\text{u}}_{i},{\left({\text{I}}_{t}-\Lambda \right)}^{-1}\Psi_{0}{\left({\text{I}}_{t}-\Lambda \right)}^{'-1}\right) \end{array}$$

However, an MTM is just-identified [24], such that changes in parametric values necessarily result in some change in the joint distribution of **y**. Conversely, SEM carries extra parameters in **Λ**, resulting in an unidentifiable likelihood function. Nevertheless, it is possible to introduce constraints in SEM to achieve parameter identifiability [24]. A constraint which is typically sufficient is coercing the residual covariance matrix **Ψ**_0 _to be diagonal, as in the examples discussed below. After defining the causal structure and achieving parameter identifiability, one may apply standard statistical methodologies (e.g., [26]) to make inferences about model parameters.

SEM models have been used to study simultaneous and recursive relationships between phenotypes in various species and breeds, such as dairy goats [27], Landrace and Yorkshire pigs [25], Holstein (e.g., [15,28-30]) and Norwegian Red (e.g., [14,31,32]) cattle. The phenotypic traits studied span from production (e.g. milk yield in dairy cattle and body weight in pigs) to reproductive (e.g. gestation length and calving ease in dairy cattle, and litter size in pigs) and health-related traits (somatic cell score and mastitis incidence in dairy cattle). In addition, some extensions of the methodology proposed by [13] have been suggested, such as threshold models with structural coefficients functioning at the level of liabilities ([15,28,33]), and models with heterogeneous structural coefficients, such as time- and yield-dependent coefficients (e.g., [15,29,31]). Some details on these applications of SEM in animal breeding and quantitative genetics are provided below.

De los Campos et al. [14,27] have presented the first applications of SEM to study recursive or simultaneous effects between traits within a quantitative genetics mixed effects models. De los Campos et al. [14] have compared four SEM specifications to study relationships between somatic cell score (SCS) and milk yield (MY) in first-lactation Norwegian Red cows using a sire model. Model parameters are estimated using maximum likelihood and the models are compared via BIC. Results indicated a recursive effect from SCS on MY, providing evidence that the negative association between MY and SCS is more likely to be due to an effect of infection (measured indirectly by the SCS) on production than to the opposite direction (i.e., a dilution effect). These results are corroborated by de los Campos et al. [27], who have studied the relationship between MY and SCS in dairy goats. The data consist of repeated measurements in each half of the udder of the animals. Again, a negative effect of SCS on MY has been observed and the evidence in favor of a dilution effect is not strong. In addition, the authors have found simultaneity of effects between SCS from the left and right halves of the udder.

Also working with MY and SCS data in dairy cattle, Wu et al. [31] have extended the simultaneous and recursive model of [13] to accommodate possible population heterogeneity. A Bayesian analysis via Markov chain Monte Carlo (MCMC) methods has been employed on test-day data of first-lactation Norwegian Red cows. Once more results suggest large negative direct effects from SCS to MY and small reciprocal effects in the opposite direction. In addition, estimated effects between MY and SCS are larger in the first 60 d of lactation than in the subsequent period, and also appear to be yield-dependent, larger in higher producing cows than in lower producing cows.

Another study concerning the relationships between MY and SCS has been conducted by Jamrozik et al. [30] with Canadian Holstein data. The authors have considered multiple-trait random regression animal models with heterogeneous (across lactations and days in milk intervals) simultaneous and recursive links between phenotypes, which are implemented using Bayesian methods via Gibbs sampling. However, in this case, model comparisons based on Bayes factors indicated superiority of simultaneous models over recursive parameterizations.

To infer simultaneous and recursive relationships between binary and Gaussian characters, Wu et al. [33] have proposed a Gaussian-threshold model within the general framework of SEM, and used such a methodology to study the relationships between clinical mastitis (CM) and MY in Norwegian Red cows. The first 180 d of lactation were arbitrarily divided into three periods of 60 days each, in order to investigate how these relationships evolve in the course of lactation. The recursive model shows negative within-period effects from (liability to) CM to MY in all three lactation periods, and positive between-period effects from MY to (liability to) CM in the following period. The results suggest unfavorable effects of production on liability to mastitis, and dynamic relationships between mastitis and test-day MY in the course of lactation.

A related application of Bayesian linear-threshold SEM has been presented by König et al. [28], who have studied the relationships between claw disorders and test-day MY in Holstein cows in eastern Germany. Four different claw disorders (digital dermatitis, sole ulcer, wall disorder, and interdigital hyperplasia) have been scored as binary traits and analyzed separately. Recursive models at the phenotypic level consider a progressive path of lagged relationships describing the influence of test-day milk yield (MY1) on claw disorders and the effect of the disorder on milk production level at the following test day (MY2). As expected, positive structural coefficients have been estimated for the gradient of disease with respect to MY1, and negative coefficients have been obtained for the rate of change in MY2 with respect to the previous claw disorder.

Other applications of Gaussian-threshold SEM with heterogeneous structural coefficients have been presented by de Maturana et al. [15,29] to explore biological relationships between gestation length (GL), calving difficulty (CD), and perinatal mortality (or stillbirth; SB) in dairy cattle. An acyclic model has been assumed, where recursive effects exist from the GL phenotype to the liabilities (latent variables) to CD and SB and from the liability to CD to that of SB considering four periods regarding GL. The results indicate that gestations ~274 days long (three days shorter than the average) lead to the lowest CD and SB levels, and confirm the existence of an intermediate optimum of GL with respect to these traits.

Working with health and fertility traits in dairy cows, Heringstad et al. [32] have employed trivariate recursive Gaussian-threshold models to analyze two fertility traits (calving to first insemination - CFI, and nonreturn rate within 56 d after first insemination - NR56) together with a disease trait, either clinical mastitis (CM), ketosis (KET) or retained placenta. The estimated structural coefficients of the recursive models indicated that presence of KET or retained placenta lengthens CFI, whereas causal effects from CM to fertility are negligible. Recursive effects of disease on NR56, and of CFI on NR56, are all close to zero. The authors conclude that selection against disease is expected to slightly improve fertility (shorter CFI and higher NR56) as a correlated response and vice versa.

Finally, Varona et al. [25] have presented an analysis of litter size and average piglet weight at birth in Landrace and Yorkshire using a standard two-trait mixed model (SMM) and a recursive mixed model (RMM). On the one hand, in Landrace, results in terms of posterior predictive model checking support a model without any form of recursion or, alternatively, a SMM with diagonal covariance matrices for all random effects considered, i.e. additive genetic, permanent and temporary environmental effects. On the other hand, in Yorkshire, the same criterion favors a model with recursion at the level of temporary environmental effects only, or, in terms of the SMM, the association between traits is shown to be exclusively due to an environmental (negative) correlation. In concluding remarks the authors suggest that the choice between a SMM or a RMM should be guided by the availability of software, by ease of interpretation, or by the need to test a particular theory or hypothesis that may be better formulated under one parameterization and not the other.

#### Recovering recursive causal structures

To fit a SEM, the matrix **Λ **of coefficients defining the causal structure must be specified. In all applications of SEM in quantitative genetics so far, the causal structure was assumed known *a priori *(e.g., [15,32]), or just a few putative structures selected using some prior knowledge were compared (e.g., [14,27,25,33]). However, it may be argued that even without information on QTL it may be possible to infer (at least partially) the causal relationships among phenotypic traits using data-driven algorithms that search for a causal structure.

For example, there are algorithms that use the notion of d-separation [3] to explore the space of causal hypotheses so as to arrive to a causal structure (or a class of observationally equivalent causal structures) that is capable of generating the observed pattern of conditional probabilistic independencies between variables. As an example, here we describe how such search can be performed for the model **y**_*i *_= **Λy**_*i *_+ **e**_*i*_.

A recursive causal structure can be represented by a Directed Acyclic Graph (DAG), which is a set of variables (or nodes) connected by directed edges (arrows). Pairs of connected nodes represent direct causal relationships. A path in the causal structure is a sequence of connected variables. Unconditionally, flows of dependence between variables in the extremes of paths may take place, unless there is a collider (variable with arrows converging at it, like *c *in *a *→ *c *← *b*) in the path. Colliders block the flow of dependency in a path, which makes *a *and *b *independent in the structure above. Conditioning on a variable that is not in the extremes of the path switches its status regarding the flow of dependence through it, i.e. if the variable is a collider it allows the flow, whereas if it is a non-collider it blocks the flow. Two variables *a *and *b *in a DAG are said to be d-separated conditionally on a subset **S **of remaining variables if there are no path between *a *and *b *such that all its nodes allow the flow of dependence (i.e., no path between *a *and *b *in a DAG such that all the colliders or its descendants are in **S **and no non-colliders are in **S**). Under some assumptions, d-separations in the causal structure of a SEM result in conditional independencies in the joint probability distribution of **y**. This is used to guide the selection of a causal structure or a class of equivalent causal structures (different causal structures that result in joint distributions presenting the same set of conditional independences) that is compatible with the joint distribution of the data [3,23].

Methodologies such as the IC algorithm [3,34] have been developed to explore the connection between recursive causal structures and joint distributions and recover underlying DAG structures (or a class of observationally equivalent structures). Based on a given correlation matrix, this algorithm performs a list of queries about conditional independencies between variables. Assuming that such independencies reflect d-separations in the underlying DAG, the algorithm returns a partially oriented graph as output, which generally results on an important constraint on the initial causal hypothesis space that could be used to fit the SEM. Partially oriented graphs are graphs with directed and undirected edges representing a class of equivalent causal structures.

Considering a set *V *of random variables, the IC algorithm can be described by the following steps:

1. For each pair of variables *a *and *b *in *V*, search for a set of variables *S*_*ab *_such that *a *is independent of *b *given *S*_*ab*_. If *a *and *b *are dependent for every possible conditioning set, connect *a *and *b *with an undirected edge. This step results in an undirected graph *U*. Connected variables in *U *are called adjacent.

2. For each pair of non-adjacent variables *a *and *b *with a common adjacent variable *c *in *U *(i.e., *a *- *c *- *b*), search for a set *S*_*ab *_that contains *c *such that *a *is independent of *b *given *S*_*ab*_. If this set does not exist, then add arrowheads pointing at *c *(*a *→ *c*← *b*). If this set exists, then continue.

3. In the resulting partially-oriented graph, orient as many undirected edges as possible in such a way that it does not result in new colliders or in cycles.

The goal of the first step of the algorithm is to obtain a graph that specifies pairs of traits that are directly connected by an edge, because variables that are adjacent in the underlying causal structure are not d-separated (hence they are not probabilistically independent) given any possible set of variables. The second step aims to orient edges by searching for unshielded colliders (structures where a collider is directly caused by two non-adjacent variables). Non-adjacent parents of a collider variable are d-separated given at least one set of variables, but not if conditioned to any set of variables that contains the collider. The observational consequence of this is the probabilistic dependence between the non-adjacent parents conditionally on every possible set of variables that contains the common child. The third step performs every further edge orienting that does not result in a new collider or in a cycle. Additional constraining of the output may be achieved by incorporating background knowledge like time precedence or other prior beliefs [4,23].

The decisions about declaring pairs of variables as conditionally dependent or not are based on partial correlations inferred from a sample, which involves some degree of uncertainty. To account for that, decisions may be made by testing null hypotheses of vanishing partial correlations or, in a Bayesian approach, using highest posterior density (HPD) intervals for the partial correlations.

The IC algorithm was developed based on the connection between causal structure and joint distribution, which requires some assumptions [23]. Maybe the strongest assumption refers to causal sufficiency: it is assumed that every variable that influences two or more variables within the set of studied variables is already within this set. In other words, it is assumed that there are no hidden causes of two or more variables. Considering that residuals in a SEM account for the sum of the effects of the parents of each trait that are not included in the model predictor, the consequence of the causal sufficiency assumption is the absence of sources of residual covariance among traits, i.e. residual covariance matrices must be diagonal [3]. However, as mentioned earlier, this model constraint (i.e., **Ψ**_0 _to be diagonal) is already adopted in recent applications of SEM in animal breeding in order to achieve model identifiability. Therefore, the assumptions of the IC algorithms are not stronger than the assumptions considered in recent application of SEM in quantitative genetics. In those applications, not only covariance matrices of random variables are assumed to be structured (usually diagonal), but the causal structure itself is assumed to be known.

#### Causal structure search within a quantitative genetics mixed models context

Valente et al. [16] have adopted a SEM setting with a diagonal residual covariance matrix, as in [14,15,32]. Within this construction, a recursive causal structure that is compatible with the joint probability distribution of the data may be searched using the IC algorithm. In the formulation described in the section above, model residuals are regarded as independent, and recursive effects are used to model (interpret) patterns of co-variability between observable variables. However, in a mixed SEM (as presented by [13]) with independent residuals, associations between observed traits are explained not only by causal links between them, but also by genetic reasons. Therefore, the unobserved correlated genetic effects considered in this context may confound the causal structure search if one tries to perform it based on the joint distribution of the phenotypes.

**Figure 5:**
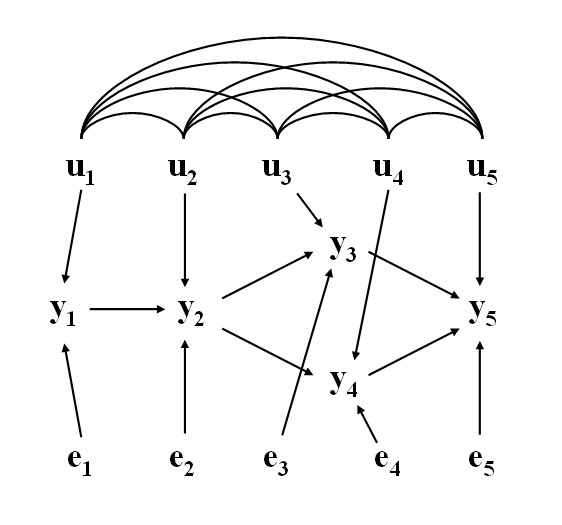
**Example of network involving five phenotypic (observable) traits, and their corresponding additive genetic (*u*'s) and residual (*e*'s) effects**. The arcs connecting *u*'s represents genetic correlations (adapted from [16])

Take as an example the causal structure depicted in Figure 5, where there are recursive relationships among phenotypes *y*_1 _through *y*_5_, with uncorrelated residuals (*e*_1_,...,*e*_5_) and correlated additive genetic effects (*u*_1_,...,*u*_5_). The connection between the causal structure among phenotypes and their joint probability distribution does not hold in a model where genetic effects are uncontrolled hidden variables. For example, given such causal structure *y*_1 _would be expected to be independent of *y*_3 _given *y*_2_, but this may not hold because of the correlation between *u*_1 _and *u*_3_.

Nonetheless, as indicated by [16], genetic relationship information between individuals gives a means of "controlling" for this confounder. Within this context, Valente et al. [16] have proposed an approach to search for acyclic causal structures in which d-separations are reflected as conditional independencies on the distribution of phenotypes after taking into account the additive genetic effects (i.e., the distribution of the phenotypes conditionally on the genetic effects). Given the model settings presented above, i.e., a SEM that accounts for additive genetic effects, the covariance matrix of the phenotypic vector **y**_*i *_can be expressed as:

$$\begin{array}{c} Var({\text{y}}_{i})={\left({\text{I}}_{t}-\Lambda \right)}^{-1}{\text{G}}_{0}{\left({\text{I}}_{t}-\Lambda \right)}^{'-1}\\ +{\left({\text{I}}_{t}-\Lambda \right)}^{-1}\Psi_{0}{\left({\text{I}}_{t}-\Lambda \right)}^{'-1}. \end{array}$$

Note that *(***I**_*t *_- **Λ***)*^-1 ^**G**_0 _*(***I**_*t *_- **Λ***)*^-1 ^and *(***I**_*t *_- **Λ***)*^-1 ^**Ψ**_0 _*(***I**_*t *_- **Λ***)*^-1 ^are the covariance matrices of additive genetic effects *(*${\text{G}}_{0}^{*}$) and of residuals *(*${\text{R}}_{0}^{*}$) obtained from a standard multiple trait mixed model that accounts for covariance between genetic effects and residuals from different traits, but not for causal relationships between phenotypes [13,25]. The covariance matrix of **y**_*i *_can be then rewritten as $Var({\text{y}}_{i})={\text{G}}_{0}^{*}+{\text{R}}_{0}^{*}$, and the covariance matrix between traits conditionally on the additive genetic effects can be represented as $Var({\text{y}}_{i}|{\text{u}}_{i})={\left({\text{I}}_{t}-\Lambda \right)}^{-1}\Psi_{0}{\left({\text{I}}_{t}-\Lambda \right)}^{'-1}={\text{R}}_{0}^{*}$. Therefore, estimates of ${\text{R}}_{0}^{*}$ can be used to select a causal structure among phenotypes.

In Valente et al. [16], the (co)variance matrix ${\text{R}}_{0}^{*}$ is inferred using Bayesian MCMC methods, in which samples are drawn from the posterior distribution of ${\text{R}}_{0}^{*}$. These samples are used then to obtain measures of uncertainty about this matrix, while accounting for uncertainty of all other parameters included in the reduced MTM. In summary, the overall statistical approach proposed by [16] consists of three stages:

1. A Bayesian MTM is fitted, and posterior samples of ${\text{R}}_{0}^{*}$ are obtained.

2. The IC algorithm is applied to the posterior samples of ${\text{R}}_{0}^{*}$ to make the statistical decisions required. Specifically, for each query about the statistical independence between variables *a *and *b *given a set of variables *S *and, implicitly, the genetic effects:

a) Obtain the posterior distribution of residual partial correlation *ρ*_*a,b*|*S*_. These partial correlations are functions of ${\text{R}}_{0}^{*}$. Therefore their posterior distribution can be obtained by computing the correlation at each sample drawn from the posterior distribution of ${\text{R}}_{0}^{*}$.

b) Compute the 95% HPD interval for the posterior distribution of *ρ*_*a,b*|*S*_.

c) If the HPD interval contains 0, declare *ρ*_*a,b*|*S *_as null. Otherwise, declare *a *and *b *as conditionally dependent.

3. Lastly, a SEM using the selected causal structure (or one member within the class of observationally equivalent structures retrieved by the IC algorithm) is fitted, as in [13], such that causal relationships (i.e., recursive effects) can be estimated.

**Figure 6:**
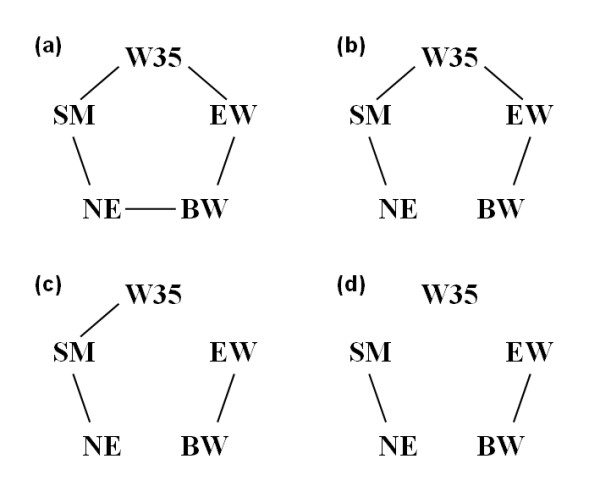
**Phenotype relationships structure recovered by the IC algorithm within a mixed model approach as described by **[16], **applied to data on meat-type quail**. Edges connecting two traits represent non-null partial correlations, as determined by Highest Posterior Density (HPD) intervals with different contents: Panel (a): 0.7, Panel (b): 0.75, 0.8, 0.85, Panel (c): 0.9, and Panel (d): 0.95; the five traits considered are BW: Birth weight, W35: Weight at 35d, SM: Age at sexual maturity (1^st ^egg), EW: Average egg weight, and NE: Rate of lay (number of eggs)

Valente et al. [16] have validated their methodology using simulated data with different causal structures and sample sizes, showing that it can indeed recover the underlying causal structure among phenotypic traits. A first application of such methodology with real data has been presented by Valente et al. [35], who have studied relationships among five traits (birth weight, weight at 35 days of age, age at sexual maturity, average egg weight, and rate of lay) in meat-type quail. The data include 854 females phenotyped for all five traits, and a pedigree file with a total of 10,680 birds. The posterior distributions of the partial correlations obtained are not very sharp, such that different HPD interval contents have been used for the statistical decisions, namely 0.7, 0.75, 0.8, 0.85, 0.9, 0.95 probabilities. Some null partial correlations have been detected; however the structures returned are completely undirected (Figure 6). In this application the edges were oriented based on time sequence information regarding the expression of each trait.

## Conclusions

Structural equation models are able to express causality among traits. However, one may fit a SEM with causal structures that do not express the actual causal relationship among traits. The inference of the causal structure is a much harder task than just describing data by a stochastic model. As discussed in this review, using the IC algorithm and related techniques involves accepting specific assumptions, from which the causal sufficiency seems to be the strongest one. In this regard, applying the IC algorithm may be regarded as a causal structure inference only if one is willing to accept the causal assumptions. Otherwise, the application of such algorithms can be viewed simply as a causal structure selection for SEM constructed with diagonal residual covariance matrices.

Nonetheless, the latter applications may still produce interesting and useful results such as the generation of causality hypotheses for further research and investigation. Such hypotheses can then be supported or dismissed by additional data collected from other studies, or they might be tested experimentally through controlled interventions. In genetics, for example, a putative causal mutation could be ultimately tested using gene knockout or knockdown methodologies. However, quite often, randomized experiments are not an alternative due to logistic or ethical constrains, and one is restricted to the analysis of observational studies. In this context, SEM and causal search tools like the IC algorithm are handy. Moreover, in genetics and genomics studies, causal inference is aided by the concept of Mendelian randomization [22], in which allelic variants are randomized to zygotes during meiosis and eventually passed on from parents to offspring, analogously to a randomized experimental design. Applying SEM-related methodologies to QTL analysis and gene mapping with multiple traits not only allows inference regarding causal relationships among phenotypes, but it also enhances detection power and precision of estimates, with the additional advantage of a distinction between direct and indirect genetic effects of QTL on each trait [12].

In addition to DNA polymorphism information and knowledge about genes or QTL that can be used as parent nodes in phenotype network reconstruction, the joint analysis of multilayer large-scale "omics" data such as transcriptome, metabolome and proteome can certainly provide added information and enhance the ability to infer causal phenotype relationships, although it also brings another level of statistical, computational and data mining challenge [36]. Moreover, structural and functional data such as gene sequence, gene localization, transcription binding sites, gene ontology, and metabolic pathway among others can also be used post hoc to verify and test putative gene and phenotype networks [36]. Such data can be used also as a priori information to aid network inference, the same way it has already been used in other "omics" applications such as microarray data [37].

SEM have also been used in the context of quantitative genetics analysis of multiple phenotypic traits when QTL or genomic information is not available [13], allowing a different interpretation of relationships among traits relative to standard multiple trait models traditionally used in animal breeding, where all relationships are represented by symmetric linear associations among traits. As discussed previously, in all applications of SEM in animal breeding so far, the causal structure was assumed known or just a few putative structures were compared. More recently, Valente et al. [16] have proposed a methodology that allows searching for recursive causal structures in the context of mixed models and quantitative genetics. Their approach involves a first step of data adjustment for genetic effects, which otherwise act as confounders of causal effects between phenotypic traits. In Valente et al. [16,35], a classical infinitesimal additive genetic model involving a relationship matrix **A **constructed from pedigree information has been considered for such task. As an alternative, if high density molecular marker data is available (e.g., SNP genotypes), more efficient genetic merit prediction approaches can be employed such as Bayesian regression techniques [38] or kernel methods [39]. This is a topic which deserves further investigation to assess the impact of better estimation of genetic effects on the ability to uncover causal links between phenotypes.

Some other areas related to phenotype network inference that would also warrant additional research refers to the development of (parametric or non-parametric) methods to deal with non-Gaussian traits, as well as search algorithms and software suitable to handle huge number (on the level of thousands) of variables. Lastly, and specifically in the context of animal and plant breeding, extra research is required to study how knowledge regarding causal effects between traits could be explored for the development of more efficient breeding programs and agricultural production enterprises.

In summary, SEM provide a flexible and insightful approach for the genetic analysis of multiple traits, allowing the characterization of pleiotropic and heterogeneous genetic effects of multiple loci on multiple traits, as well as causal relationships among phenotypes, which can be used to predict behavior of complex systems, e.g. biological pathways underlying disease traits. More specifically with livestock, SEM can be used to infer phenotype networks in the genetic analysis of quantitative traits, such that the effect of external interventions can be better predicted. This may foster the development of more efficient breeding programs and optimal decision-making strategies regarding farm management practices.

## Competing interests

The authors declare that they have no competing interests.

## Authors' contributions

GJMR and BDV wrote the manuscript; GC, XLW, DG and MAS provided critical insights and helped revising the manuscript. All authors read and approved the final manuscript.
